# How participation in surgical mortality audit impacts surgical practice

**DOI:** 10.1186/s12893-017-0240-z

**Published:** 2017-04-19

**Authors:** Chi-Wai Lui, Frances M. Boyle, Arkadiusz Peter Wysocki, Peter Baker, Alisha D’Souza, Sonya Faint, Therese Rey-Conde, John B. North

**Affiliations:** 1 School of Public Health, Faculty of Medicine, University of Queensland, Herston Road, Brisbane, QLD4006 Australia; 2 Department of Surgery, Logan Hospital, Logan City, Queensland Australia; 3 Queensland Audit of Surgical Mortality, Royal Australasian College of Surgeons, Brisbane, Queensland Australia

## Abstract

**Background:**

Surgical mortality audit is an important tool for quality assurance and professional development but little is known about the impact of such activity on professional practice at the individual surgeon level. This paper reports the findings of a survey conducted with a self-selected cohort of surgeons in Queensland, Australia, on their experience of participating in the audit and its impact on their professional practice, as well as implications for hospital systems.

**Methods:**

The study used a descriptive cross-sectional survey design. All surgeons registered in Queensland in 2015 (*n* = 919) were invited to complete an anonymous online questionnaire between September and October 2015. 184 surgeons completed and returned the questionnaire at a response rate of 20%.

**Results:**

Thirty-nine percent of the participants reported that involvement in the audit process affected their clinical practice. This was particularly the case for surgeons whose participation included being an assessor. Thirteen percent of the participants had perceived improvement to hospital practices or advancement in patient care and safety as a result of audit recommendations. Analysis of the open-ended responses suggested the audit experience had led surgeons to become more cautious, reflective in action and with increased confidence in best practice, and recognise the importance of effective communication and clear documentation.

**Conclusions:**

This is the first study to examine the impact of participation in a mortality audit process on the professional practice of surgeons. The findings offer evidence for surgical mortality audit as an effective strategy for continuous professional development and for improving patient safety initiatives.

## Background

The surgical mortality audit is a systematic review process to examine issues relating to the causes of death in patients following surgery. Audit trends in surgical mortality are subsequently documented which helps to identify errors or gaps in surgical management and patient care [1–6]. Such auditing processes, especially those that involving peer-review procedures, should result in improved patient outcomes, quality of service and reflective practice in surgical care settings. Previous research has shown that the implementation of surgical auditing is associated with improved patient care and clinical outcomes [7, 8] as well as reduction of healthcare costs at the hospital level [9, 10].

Even though surgical mortality audit is an important tool for quality assurance and professional development, relatively little is known about the impact of participation in such a review process on professional practice at the individual surgeon level. Specifically, it is unclear what lessons have been learnt through participating in the mortality audit and to what extent the experience of receiving feedback from peers, or being involved in the peer review process with other surgeons, affects the clinical practice of these surgeons. This study addresses this gap in the evidence-base by analysing answers to an audit quality assurance survey provided by a sample of surgeons in Queensland, Australia in 2015. The study focused on their experience of participating in the audit process and its impact on their professional practice, as well as implications for hospital systems. The findings deepen our understanding of the experiences and impacts of participation in surgical mortality audit and help identify areas for further improvement of this peer review process.

### Surgical mortality audit in Queensland

Australia is one of the few countries that implement a nationwide audit of surgical deaths. The Australian and New Zealand Audit of Surgical Mortality (ANZASM) provides a national framework of audits of surgical mortality using a peer-review procedure [11]. In Queensland, the Queensland Audit of Surgical Mortality (QASM) is funded by Queensland Health and operates under the governance of the Royal Australasian College of Surgeons. QASM administrates and manages the audit and disseminates the audit results. As at April 2017, there are 29 public hospitals and 39 private hospitals participating in QASM [12].

Participation in surgical audit for surgeons in Queensland is a requirement of the Continuing Professional Development Program of the Royal Australasian College of Surgeons. The surgeons may participate in QASM in three ways: submitting a structured Surgical Case Form (SCF) for each death that occurs in a patient under their care; acting as a first-line assessor; and/or acting as a second-line assessor. The audit process involves QASM sending a SCF to the consultant surgeon whose patient died in the hospital regardless of whether an operation was performed or not. The completed form undergoes a blinded (for patient, surgeon and hospital) first-line assessment. If the assessor is satisfied that there were no issues requiring further in-depth assessment, then the case is closed and feedback sent to the surgeon involved. If further assessment is recommended or information on the case is lacking, the medical record of the patient for that admission is acquired and a second-line assessor within the same surgical speciality examines the SCF, the first-line assessor’s comments, and the patient’s medical record before compiling a final report to QASM. This feedback of the second-line assessor is then passed on in a de-identified document to the original surgeon under whose care the patient died. Between 2007 and 2015, a total of 7,444 deaths were reported to QASM, 100% underwent first-line assessment and 909 or 12% of these went to second-line assessment [13].

The Audits of Surgical Mortality are protected by Commonwealth legislation known as ‘Qualified Privilege’. This ensures comprehensive privacy regarding the data at all levels and is key to the cooperation that is demonstrated by surgeons across Queensland and the nation. The individual surgeon receives feedback on his/her patient after assessment but no other party is able to access this information under the Qualified Privilege legislation. Aggregated de-identified data may be used in publications, presentations or other teaching materials.

## Methods

### Survey design and participants

This study used a descriptive cross-sectional design to survey a self-selected cohort of surgeons about their experiences when participating in the surgical mortality audit. The researchers designed a short anonymous online questionnaire instrument using SurveyMonkey [14] and all surgeons registered to work in Queensland in 2015 (*n* = 919) were invited to complete the survey between 18 September and 23 October 2015. The use of an online survey was deemed the most effective for the target population.

The questionnaire used closed questions to obtain data on the surgeon’s speciality and nature of their participation in the QASM process: no participation; submitted at least one SCF; conducted at least one first-line assessment; conducted at least one second-line assessment. For each form of participation, surgeons were asked whether they thought the feedback they had received or provided had influenced their own clinical practice. The surgeons were also asked whether they had perceived any changes to hospital practices as a result of QASM recommendations. Open-ended questions invited surgeons to comment on the impact of participation within the surgical audit regarding clinical and hospital practices as well as the perceptions and recommendations that arose from experiences with the peer review procedures.

All questionnaire responses were transferred into an Excel worksheet for further analysis.

### Data analysis

Descriptive statistics were used to summarise details of participation of the respondents in the surgical audit and to compare the perceived impacts of participation across surgical speciality, the number of participants in auditing, and nature of participation (as surgeon reporting or assessor) in the peer review process. To examine the relationship between the level of participation in surgical audit and influence of feedback or assessment on clinical practice, a chi-square analysis was conducted [15]. The chi-squared test was appropriate for this study as the data met the assumption that the expected frequency of any cell was at least five.

The open-ended survey responses on experiences and perceived impacts of auditing were coded independently by two researchers (CWL, FMB) using an inductive thematic analysis approach [16]. The themes identified were compared with differences resolved through discussion between the two researchers. The findings were then presented to the full research team for further examination.

## Results

The link to the survey was sent by email to 919 surgeons in Queensland and 184 surgeons completed and returned the questionnaire: a response rate of 20%. Table 1 shows that the distribution of surgical speciality of the survey participants is closely comparable to that of the wider population of surgeons working in Queensland in 2014.

**Table 1:** Number and Surgical Speciality of Participants

Surgical Specialty	No.	%	Distribution of Active Surgeons in Queensland 2014 (%)^a^
Cardiothoracic Surgery	5	2.7	4.2
General Surgery	61	33.3	31.4
Neurosurgery	10	5.5	4.8
Orthopaedic Surgery	41	22.4	28.8
Otolaryngology Head & Neck Surgery	19	10.4	9.3
Paediatric Surgery	7	3.8	1.5
Plastic & Reconstructive Surgery	12	6.6	7.2
Urology	17	9.3	9.1
Vascular Surgery	12	6.6	3.7
*Total*	*184*	*100*	*100*

^a^ Source: Royal Australasian College of Surgeons, *Activities Report, 1 January to 31 December 2014*, p37.

### Extent of participation in the QASM process

Most respondents (*n* = 149; 81%) reported having participated in the QASM process by completing at least one form of QASM activity. The 35 respondents who stated they had not participated in the QASM process are not included in the analysis that follows. Submitting a SCF was the most common type of participation, followed closely by being a first-line assessor: 117 (79%) surgeons reported they had completed at least one case form and 107 (72%) reported having completed at least one first-line assessment. Just over half (*n* = 80; 54%) of the surgeons reported having participated as a second-line assessor.

Considerable individual variation was evident among the 149 respondents with regard to the degree of participation in the QASM process (see Table 2). At the lowest level, 35 (23%) participants reported submitting a SCF only, with no involvement in first or second-line assessments. These were the surgeons who had received but had not provided feedback in the QASM process. At the other end of the spectrum were 55 (37%) surgeons who reported participation in all three components of the QASM process, i.e. they had submitted at least one case form, and had been involved in both a first and second-line assessment. There were other intermediate levels of participation along this spectrum of participation (refer to Table 2).

**Table 2:** Percentages of surgeons reporting whether QASM influenced their clinical practice according to level of QASM participation

Type of Participation	No. of responses	Feedback influenced Clinical Practice			Assessment influenced Clinical Practice		
		Yes	No	Total	Yes	No	Total
		n (%)	n (%)	n (%)	n (%)	n (%)	n (%)
SCF Only^a^	35	2 (6)	33 (94)	35 (100)	-	-	-
Assessment Only^a (FLA, SLA or both)^	32	-	-	-	13 (41)	19 (59)	32 (100)
SCF & Either First or Second-line Assessment	27	8 (30)	19 (70)	27 (100)	10 (37)	17 (63)	27 (100)
SCF & Both Assessments	55	12 (22)	43 (78)	55 (100)	28 (51)	27 (49)	55 (100)
Total	149	22 (19)	95 (81)	116 (100)	51 (45)	63 (55)	114 (100)

Statistical comparison of the influence of feedback on clinical practice according to the level of participation:

Chi-squared = 6.33 (df = 2), *p* = 0.04

Statistical comparison of the influence of assessment on clinical practice according to the level of participation:

Chi-squared = 1.71 (df = 2), *p* = 0.42

*SCF* surgical case form, *FLA* first-line assessment, *SLA* second-line assessment

^a^ Influence of assessment not applicable to surgeons who completed SCF only; influence of feedback not applicable to surgeons who completed Assessment only

### Impacts of QASM participation on clinical and hospital practices

Thirty-nine percent (*n* = 58) of the 149 surgeons who had participated in QASM activity reported that their involvement had affected their clinical practice. As seen in Table 2, reported impacts on clinical practice were more commonly a consequence of assessment completion than receiving feedback. Only 2 (6%) of the 35 participants whose participation was confined to receiving feedback through SCF submission reported impacts on clinical practice compared with 22–30% of those who completed assessments in addition to receiving feedback (*p* = 0.04).

20 participants (13%) also reported that they perceived changes to hospital practices as a result of QASM recommendations (detailed results not shown). The analysis of open-ended responses below provided further details of the impacts on clinical and hospital practices.

### Analysis of open-ended responses

Table 3 reports the major themes and their frequency as identified from analysis of answers to open-ended survey questions. Fifty-nine participants described how participation affected their clinical practices: ‘becoming more reflective by learning from experiences of peers’ and ‘becoming more considered in their actions or decisions’ being the two most frequently mentioned. Participants also thought that the audit experience helped them recognise the importance of effective communication and clear documentation as well as increased their confidence in understanding and implementing best practice. In addition, there were reports on changes in specific clinical technique or procedure including ‘altered antibiotic prophylaxis’, ‘use of stents’, or be ‘more conscious of fluid balance’ and ‘avoiding anticoagulants’.

**Table 3:** Major Categorised Themes, Examples, and Frequencies of Open-ended Survey Responses to Questions on Clinical and Hospital Practices

Major Categorised Theme	Example	Reply No. (%)
Impacts on Clinical Practice		*n* = 59
Be more reflective by learning from mistake/approach of others	‘Gives you clinical situations to consider, and put yourself in same position. What would I do differently.’ ‘I reflect on how I might have managed the patient and put myself in their shoes when I do the assessment.’ ‘It has made me more aware of how to try and avoid problems within my field of practice.’	24 (41%)
Become more considered in action or decision- making	‘I try to make sure all bases covered and all contingencies have been considered before I choose a course of action.’ ‘In one instance it prompted me to do follow up radiology routinely rather than only when there were worrying symptoms.’ ‘Acts as a reminder to be careful regarding pre-operative preparation.’	13 (22%)
Recognise importance of effective communication and clear documentation	‘Ensured I made verbal handovers to fellow consultants to ensure subtle points were not lost via ‘registrar to registrar’ handover.’ ‘Doing assessments emphasises the importance of early consultation with consultants and clear.’ documentation of consultant input.’ ‘Reminded me of the importance of informative and legible notes.’	7 (12%)
Increase in confidence on practice	‘Useful information to take into consideration for future cases. Also useful to know that an independent reviewer finds that your care had no concerns etc. in their opinion.’ ‘Useful to be reassured re practice.’ ‘Feel reassured.’	4 (7%)
Impacts on Hospital Practice		*n* = 19
Improve procedure and patient management (organisation level)	‘Increased access to theatres for emergency cases.’ ‘Greater focus on avoiding delays in treatment as delays are repeatedly shown to have a negative impact on outcomes.’ ‘Encouragement of better written notes by consultants.’	10 (53%)
Improve patient care and safety (theatre level)	‘More consultant leadership on surgical units.’ ‘More attention to detail where deaths have occurred and more stringent protocols in the OT [operating theatre].’ ‘More vigilant in non orthopaedic care of morbidities that have potential to influence the outcome of orthopaedic surgery.’	4 (21%)
Changes recommended for Hospital Practice		*n* = 40
Better audit process and feedback use	‘Only selected centres should be entitled to undertake some procedures a robust MDT [multi-disciplinary team] + M/M [morbidity and mortality] + Audit is required in centres wishing to undertake complex surgeries.’ ‘The distribution of feedback is not clear to me. I have seen situations where changes to a hospital system would seem desirable but I do not know if that ever is fed back.’ ‘All hospitals should have a VMO [visiting medical officers] committee which includes a physician, surgeon, oncologist, anaesthetist and intensivist to review all inpatient deaths.’	11 (28%)
Better consultant involvement and leadership	‘In clearly complicated cases, it would be appropriate for senior consultants to give an opinion on care and management of cases particularly in cases of advanced malignancy when palliative care may be more appropriate than operative intervention.’ ‘In regional hospitals, mandated earlier consultant involvement in unwell patients; by which I mean having the consultant physically attend the patient.’ ‘Consultant should be in OT [operating theatre] for all [patients] take back to theatre for [due to] complications (not just a reg [registrar] alone).’	11 (28%)
Improve procedure, facilities and training	‘Simplifying referral pathways.’ ‘Continuity of care. Access to emergency operating in normal hours.’ ‘Junior doctors need to understand fluid physiology.’	6 (15%)
Improve management of frail or elder patients	‘Better assessment and triage of frail patients for whom surgical intervention would be futile.’ ‘Dedicated NOF [neck of femur] lists or priority for elderly fracture patient reduces morbidity and mortality- and reduces bed stay and surgeon frustration. This should be supported in every Queensland Hospital.’ ‘Falls prevention in hospitals needs to be more than just filling in a form and moving their bed closer to the desk.’	5 (13%)
Improve communication and documentation	‘Accurate documentation in M&M [morbidity and mortality] meetings.’ ‘Two step change. Firstly establish an ASU [acute surgical unit] and secondly establish compulsory consultant to consultant communication protocols.’ ‘More detailed notes on treatment and discussions with colleagues and families and more second opinions!’	3 (8%)

Nineteen participants (13%) had perceived a number of changes to hospital practice as a result of QASM recommendations. Most of the changes observed were either improvement in procedure and patient management at the organisation level or advancement in patient care and safety at the operating theatre level. Based on their experiences with the QASM process, 40 participants suggested changes that could be made to hospital systems. These recommendations fall into a number of areas with ‘better audit process and feedback use’ and ‘better consultant involvement and leadership’ as the most frequently mentioned topics followed by improvements in the areas of ‘procedures, facilities and training’, ‘management of frail or elderly patients’, and ‘communication and documentation’.

Nineteen participants (13%) reported that they perceived negative effects of the QASM process (detailed results not shown). There were concerns about over-auditing and creating extra workload to the staff that is ‘repetitive and wasteful’. Participants also complained about the poor quality of advice received or that the result of an assessment was not properly followed up by the administration. Defensive practice was another negative effect highlighted in the responses with participants mentioning a reluctance to ‘operate [on] complex cases’ or ‘offer surgery to older/unwell patients who have a significant chance of potential benefit but not insignificant risk’.

## Discussion

To our knowledge, this is the first study to examine the impact of participation in a mortality audit process on professional practice at the individual surgeon level. In summary, surgeons who played an active role benefited the most. It appears that surgeons that passively participated in the audit process by filling in the SCF missed out on an educational opportunity. The often-cited aphorism ‘Good judgment comes from experience; experience comes from bad judgment’, which was attributed to Dr Kerr White [17], helps illustrate this point: a ‘bad’ surgeon is not the one with patients whose outcome was undesirable but the surgeon who lacks a process which allows them to maintain sound judgment. Participation in assessment in the surgical audit is one way of becoming a proficient surgeon, who employs critical thinking as part of their daily practice.

Just over one-third (*n* = 58) of the participants in the survey reported that involvement in the QASM process affected their clinical practice and this was particularly the case for surgeons whose participation included assessment. In addition, 13% or 20 of the participants perceived improvement to hospital practices or advancement in patient care and safety as a result of QASM recommendations. Analysis of the open-ended responses suggested the audit experience had led surgeons to become more cautious, reflective in action and with increased confidence in best practice, and recognise the importance of effective communication and clear documentation. In combination, these findings offer evidence for surgical mortality audit as an effective strategy for continuous professional development and for improving patient safety initiatives.

Our finding that the extent of participation in the audit process is associated with improvements in clinical practice is revealing. In particular, it highlights the experience of being a peer-assessor as an important learning opportunity for the surgeons. This point is clearly illustrated in the open-ended responses where participants repeatedly mentioned how seeing the work or approach of their peers helped increase their practice confidence as well as stimulated critical and reflective thinking. The greater impact of assessment activity on clinical practice other than receiving feedback from SCF is understandable. Being an assessor, especially a second-line assessor, means that the surgeon has access to comprehensive information regarding the case under review, providing opportunity and resources for learning and reflection.

The finding that submitted case forms had a low impact on clinical practice suggests that receiving feedback alone may not be a useful learning tool for surgeons. Anecdotally it has long been known that for some surgeons compliance to SCF completion is low with a delegation of the task to a registrar whose knowledge of patients or nuances of the case may be limited. The results indicate that a more detailed study to examine how surgeons perceive and respond to feedback may be warranted. There is also a need for QASM to refine and improve incident reporting and feedback procedures to hospitals.

A number of limitations associated with the cross-sectional survey design are acknowledged. First, the low response rate (20%) to the survey may have biased the results. The low response rate of surgeons to the survey is not unexpected as health professionals with their very demanding work schedules are known to be a particularly hard-to-reach group in surveys with response rates often lower than that of the general population [18]. Notably, the distribution of the participants according to surgical speciality was comparable to that of the population of surgeons working in Queensland, suggesting that the study captured a variety of opinions across specialities. However, being based on a self-selected sample, it is possible that the survey results may reflect the views of surgeons with a particular interest in the topic. In addition, while participation in the surgical mortality audit had been made mandatory for all surgeons by the Royal Australasian College of Surgeons since January 2010, participation in the reviewing of cases remains voluntary. The fact that over half of the survey participants completed first and second-line assessment indicates that the study may have attracted surgeons who are more open to and engaged with the goals of the audit process. To reduce nonresponse bias, future studies may consider using gift and professional development incentives, registered mail, reply paid envelope or a mix of web and mail survey to increase the response rate of this target group [19–21].

Moreover, as a way to keep the survey completion time short, the questionnaire included only blunt measures that collected no specific details of QASM activities (including the total number of assessments conducted by each participant). It was also not possible for the researchers to follow up or clarify meanings with the respondents regarding open-ended answers. Finally, all data reports were self-reported and the interval between participation in QASM activity and survey completion varied for individual surgeons, both of which raise the possibility of recall errors.

In combination, the results of the survey both highlight and reinforce the idea that the conduct of mortality audit needs to be an ongoing continual improvement process. As Leistikow and colleagues [22] concluded in their review on the practice of healthcare incident reporting, the ‘journey’ (i.e. the process of seeing and learning) may be more important than the ‘arrival’ (i.e. obtaining feedback or a particular outcome). The potential value of assessment activity on clinical practice reported by surgeons in this study helps illustrate this point. At the same time, the findings of the survey highlight the need for auditing bodies like QASM to take further actions to address negative impacts or disadvantages of clinical audit [23], which include creating extra workload, the perception of surveillance or territorial suspicions. In particular, it is essential to find ways to counter the practice of ‘defensive’ medicine as an unintended consequence of mortality audit. To achieve the intended goals of clinical audit, reporting systems must be underpinned by effective learning processes with a genuine focus on improved care systems rather than punitive action that intensifies feelings of shame and anxiety in the individuals [22]. Harnessing the potential of clinical audit to enhance reflective practice is a key element of that process.

## Conclusions

By focusing on how participation in the mortality audit process affects professional practice, this study shows the importance of mortality audit in helping surgeons to reflect critically on their experiences and to engage in a process of continuous learning. The findings that active involvement in the peer review procedure is associated with improvements in clinical practice confirm surgical mortality audit as an effective strategy for quality assurance and professional improvement. The study deepens our understanding of the impacts of the audit process at the individual surgeon level while contributing to the evidence-base of lifelong learning and continuing professional development.

## Abbreviations

ANZASM: Australian and New Zealand Audit of Surgical Mortality
QASM: Queensland Audit of Surgical Mortality
SCF: Surgical Case Form
